# Giant cerebral tuberculoma mimicking tumor in a pediatric patient: A case report

**DOI:** 10.1016/j.radcr.2024.08.083

**Published:** 2024-09-17

**Authors:** Muhamad Aufa Ni'ami, Dian Nurhayati

**Affiliations:** Radiology Department, Faculty of Medicine, Universitas Airlangga-Dr. Soetomo General Academic Hospital, Jalan Mayjen. Prof. Dr. Moestopo 47, Surabaya, East Java, 60131, Indonesia

**Keywords:** CNS TB, Intracranial tuberculoma, Extrapulmonary TB, Brain MRI

## Abstract

Tuberculosis (TB) is an infection caused by *Mycobacterium tuberculosis*, an infectious disease endemic in developing countries. Indonesia is ranked second only to India in terms of TB incidence in the world. TB generally manifests in the respiratory system, which can then spread hematogeneously or lymphogeneously to extrapulmonary organs. Intracranial tuberculoma is a rare manifestation of TB when compared to the overall TB presentation. Central nervous system involvement ranges from 2-5% and increases to 15% in cases of AIDS-related TB, with the percentage of tuberculoma findings around 1% in other intracranial TB cases. The most common manifestation is tuberculous meningitis. Central nervous system (CNS) involvement is a severe manifestation of TB, with high mortality and neurological morbidity. In this case report, the author presented a 6-year-old girl with giant cerebral tuberculoma, which, at the time of surgery, resembled a neoplasm with a nonspecific history of TB. MRI can visualize abnormalities with specific characteristics; Clinically and radiologically, CNS TB can mimic other infections or noninfectious conditions such as neoplasms. Therefore, clinicians can take appropriate management actions in order to prevent mortality and disability due to sequelae in CNS TB cases.

## Introduction

Tuberculosis (TB) remains one of the world's health problems. Based on estimates by the Indonesian Ministry of Health, approximately 1.06 million new cases will occur in 2023 in Indonesia. TB not only affects the lungs but also affects extrapulmonary organs, with an incidence of 8% of all TB cases in Indonesia. One of the severe manifestations that must be considered is TB of the central nervous system (CNS TB). This type of TB is correlated with high morbidity and mortality rates. Tuberculoma is a form of CNS TB that is rare and complex to diagnose. TB often occurs in developing countries, especially in Asia (58%) and Africa (28%), with Indonesia ranked second in the world [1].

TB is a case of infection caused by *Mycobacterium tuberculosis* (acid-resistant bacilli) with an airborne spread pattern through droplets from patients when coughing or sneezing. The lungs are the primary affected organs, though they can also affect the musculoskeletal, abdominal, and central nervous systems (CNS). TB infection is also exacerbated by immune system disorders in patients with acquired immunodeficiency syndrome (AIDS) and the emergence of multi-drug-resistant TB (MDR TB). In 2021, it was estimated that the number of deaths from TB would reach 1.6 million, with 187,000 constituted by TB with AIDS, which includes 11% of children with AIDS [1,2].

There are 2 types of CNS tuberculosis, namely meningeal tuberculosis and parenchymal tuberculosis with the pattern type list in Table 1, in which the intracranial tuberculoma will be discussed explicitly in this case. The diagnosis can be made with a multidisciplinary approach, with the MRI of the head playing a paramount role in the imaging, with and without contrast; however, there are times when diagnostic imaging cannot establish the main cause due to the atypical picture of TB on diagnostic imaging [3,4].

**Table 1:** Pattern of findings in CNS TB cases [3].

Meningeal tuberculosis	Parenchymal tuberculosis
Leptomeningitis	Tuberculoma
Pachymeningitis	Cerebritis
	Abscess
	Rhombencephalitis
	Encephalopathy

Intracranial tuberculoma is a granulomatous mass resulting from the hematogenous spread of tuberculosis infection. It is the result of a conglomeration of tubercular microgranulomas invading the gray-white matter junction due to hematogenous dissemination of bacteria accompanied by decreased vascular caliber in the infected area [3,4].

Tuberculomas are usually confirmed by MRI examination in patients with suspicion of central nervous system involvement. The assessment is performed using intravenous contrast with conventional or advanced MRI examinations; typically, the picture that will be found is granuloma with central caseous necrosis. Clinically, they are presented with headaches, seizures, fever, and neurologic deficits with signs of increased intracranial pressure [3,4].

## Case report

A 6-year-old girl visited our hospital with left limb weakness approximately 1 year ago, with the initial complaint of the right ear discharge with rancid and yellowish liquid. She also reported a history of recurrent fever, with no history of seizures or prolonged cough. TB contact and a previous TB infection were denied. The patient was seeking treatment for this complaint for the first time.

**Fig. 1:**
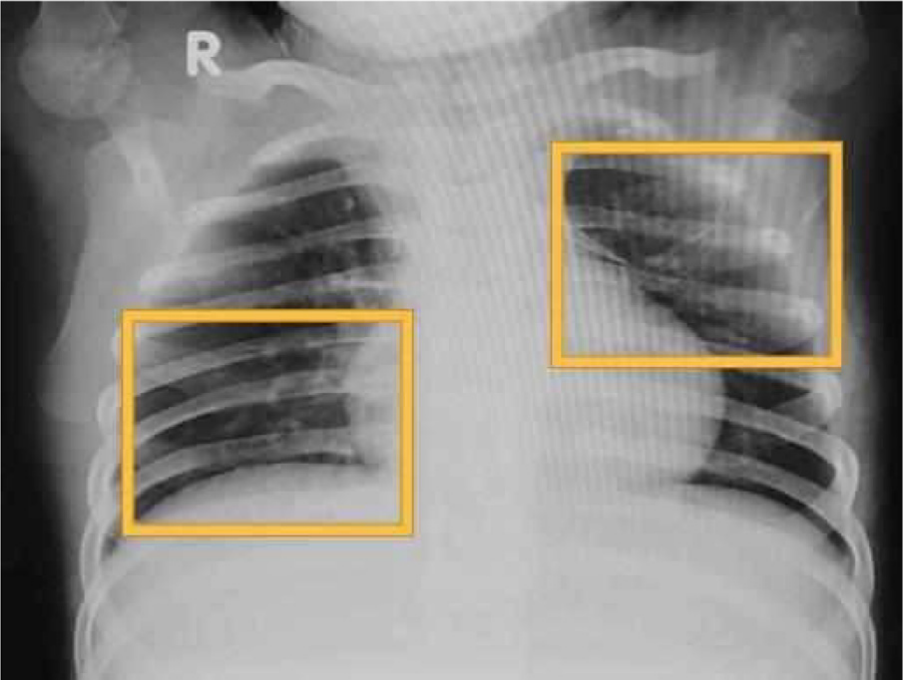
Infiltrates in the right perihilar-paracardial and left supra hilar para hilar may represent pneumonia.

The results of the CXR examination can be seen in Fig. 1; although there was no typical picture of pulmonary TB, the presence of TB still could not be ruled out.

**Fig. 2:**
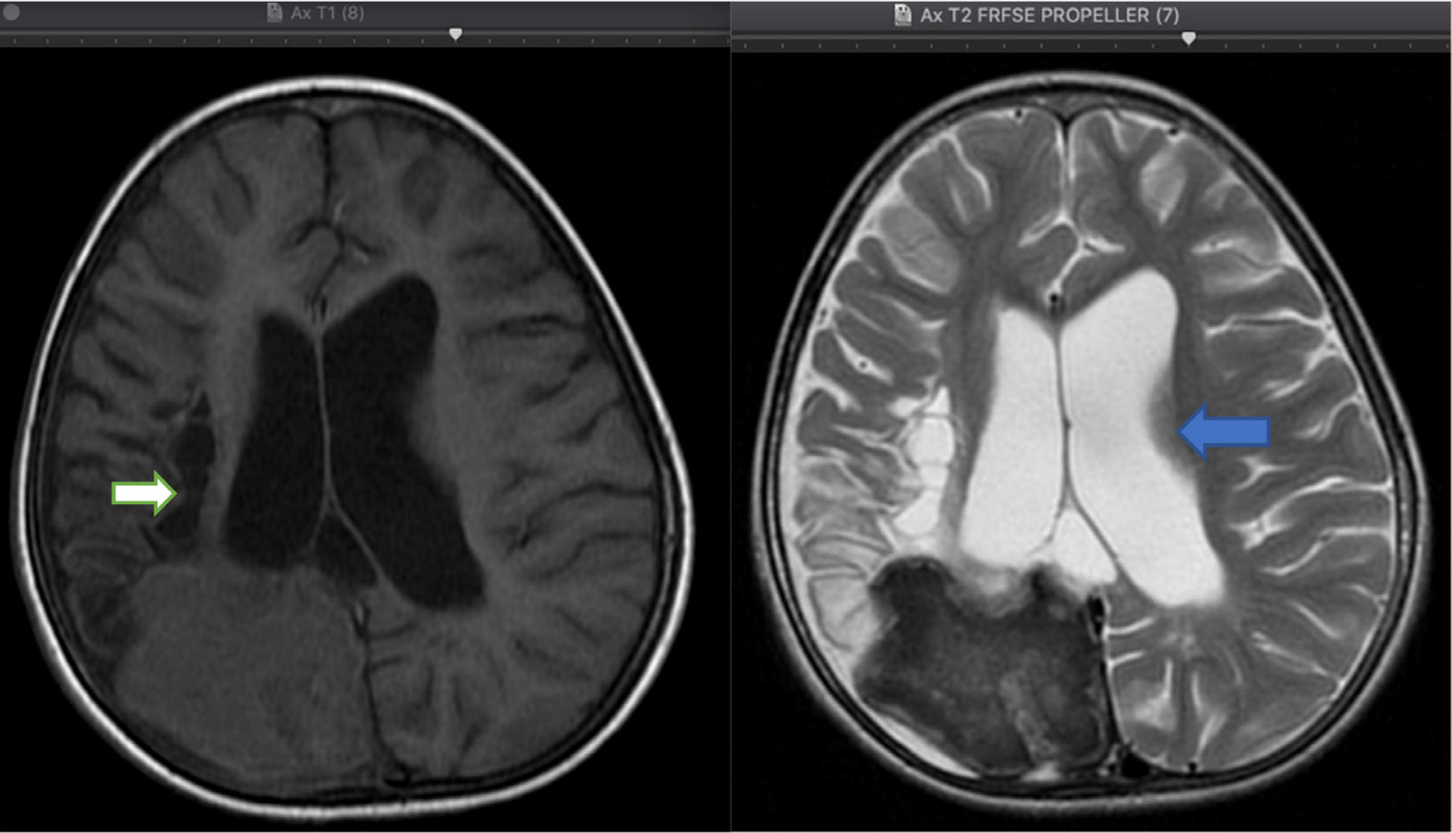
The axial section of the TI and T2 MRI shows multiple cystic lesions in the subcortical of the right parieto-occipital lobe representing *encephalomalacia cyst* (white arrow) with communicating hydrocephalus of the right and left lateral ventricles (blue arrow).

A contrast-enhanced MRI scan of the head was performed, revealing a picture of pachymeningeal enhancement in the right temporo-parieto-occipital region, with the enhancement in the left cysterna basalis and rim contrast enhancement in the cortical-subcortical lesion of the right occipital lobe with may represent meningoencephalitis with infected cystic lesion see Fig. 2. These findings strongly suggest tuberculoma with a picture of an encephalomalacia cyst and communicating hydrocephalus

Based on the result of contrast-enhanced head MRI, the patient was referred to neurosurgery for open biopsy and wide excision of the mass. A mass formation was found in the right occipital lobe with the impression of tuberculoma and a differential diagnosis of neoplasm. After that, the biopsied mass was handled by an anatomical pathologist for hematoxylin eosin (HE) staining with the final result confirming the granulomatous inflammation to be consistent with tuberculosis.

## Discussion

Pediatric patients and young adults are often the age at which CNS TB cases are most likely to occur. Gender does not affect the distribution of the disease. The main complaints include headaches, seizures, and progressive motor deficits, which may also be accompanied by other signs of increased intracranial pressure. In accordance with this case report, our patient presented a similar pattern of symptoms [5].

In pediatric patients, the classic picture of cavities or consolidation in the upper zone of the lung is not obvious in many cases. Chest X-ray (CXR) radiological findings that are often found in children are hilar or mediastinal lymphadenopathy. However, these results need to be proven by other supporting examinations, such as tuberculin tests or sputum smears from gastric lavage or sputum [2,6].

In 60% of TB patients with normal CXR, lymphadenopathy is more common on CT scans of the thorax without contrast with a characteristic of hypodense central node surrounded by calcification or *rim contrast enhancement,* sometimes accompanied by perinodal *fat* obliteration. Miliary TB can also be found in pediatric patients, especially in *immunocompromised* patients. The role of CXR *imaging* in TB is for diagnosis, therapeutic response follow-up, and detection of complications in primary care, along with ultrasound, while the use of CT and MRI is concentrated in tertiary care, especially in cases with a real suspicion of complications [6,7].

Central nervous system TB (CNS TB) is a severe manifestation of TB, causing morbidity and mortality in pediatric patients, with 25% mortality and 66% disability. CNS TB is usually caused by hematogenous spread. Clinically and radiologically, CNS TB can mimic other infections as well as other noninfectious conditions such as neoplasms [6,8].

The typical radiologic findings of the TB neuroradiologic triad are basal meningeal *enhancement*, hydrocephalus, and infarction. In other literature, the classic findings are TB meningitis, hydrocephalus, or tuberculoma. Locations other than the cisterna basalis that are often infected are the cerebellar tentorium, the area around the optic chiasm, the Sylvian fissure, and the ependymal and choroid plexus. Furthermore, MRI is superior in detecting basal enhancement because CT scans are often camouflaged with vascular enhancement in these areas [6,9].

**Fig. 3:**
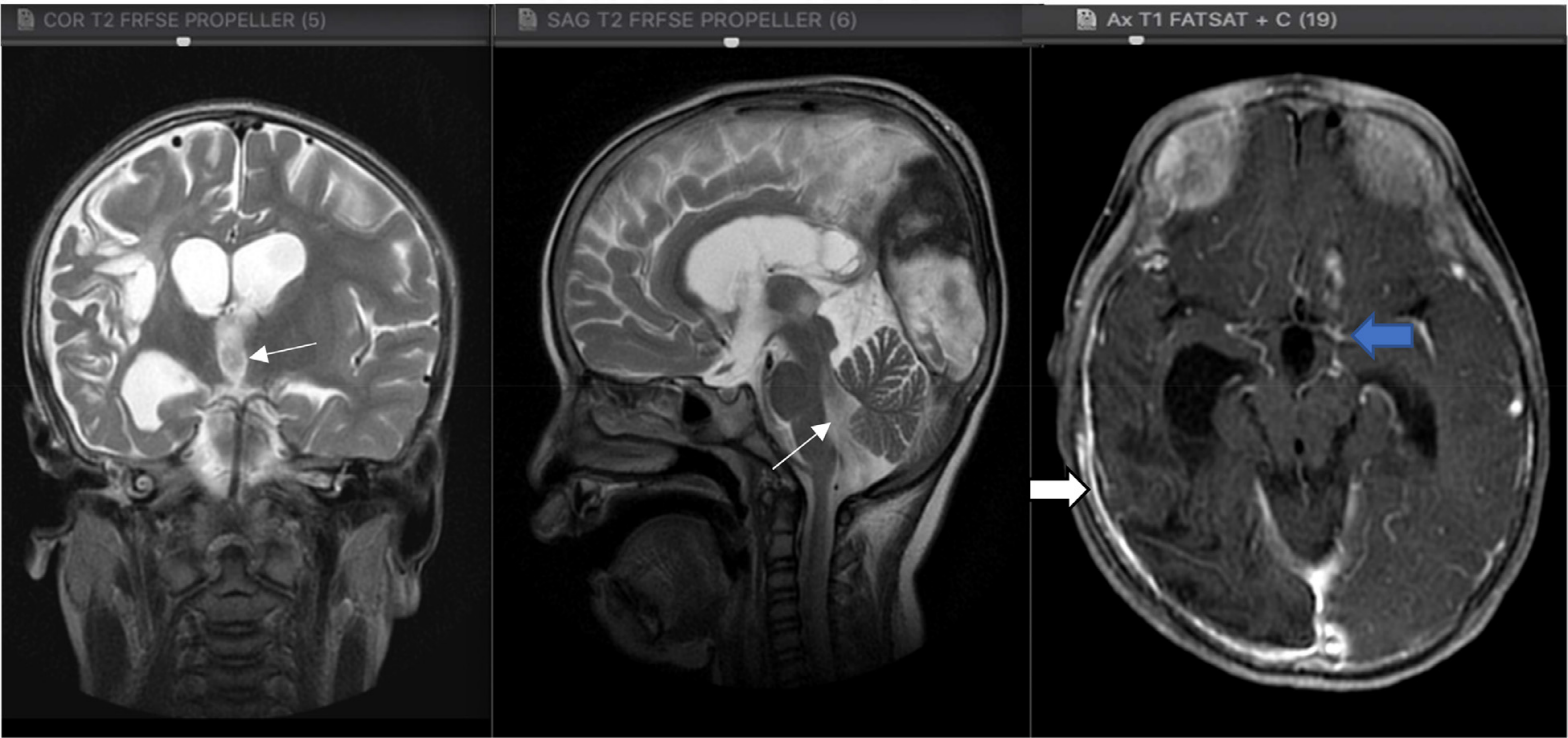
The coronal and sagittal section of the T2 MRI, dilation of the III and IV ventricles (white thin arrow) with pachymeningeal enhancement in the right temporo-parieto-occipital region (white arrow), and from axial section of the T1 fatsat MRI, the enhancement in the left cisterna basalis on contrast administration (blue arrow).

The above description is in line with our case, as our MRI examination found multiple encephalomalacia cysts, which could be the result of infarction or infection sequelae as well as *communicating hydrocephalus*. The finding of *communicating hydrocephalus* might be due to obstruction of cerebrospinal fluid absorption by inflammatory exudates in the subarachnoid cisterna basalis or due to secondary narrowing of the aqueduct or ventricle given the focal lesions in the parenchyma and mass squeeze effect by meningeal exudates, mesencephalic edema or exudates in the intraventricular. In our case, inflammation in the cisterna basalis was felt to be more suitable, as per the findings of Fig. 3, Fig. 6.

**Fig. 4:**
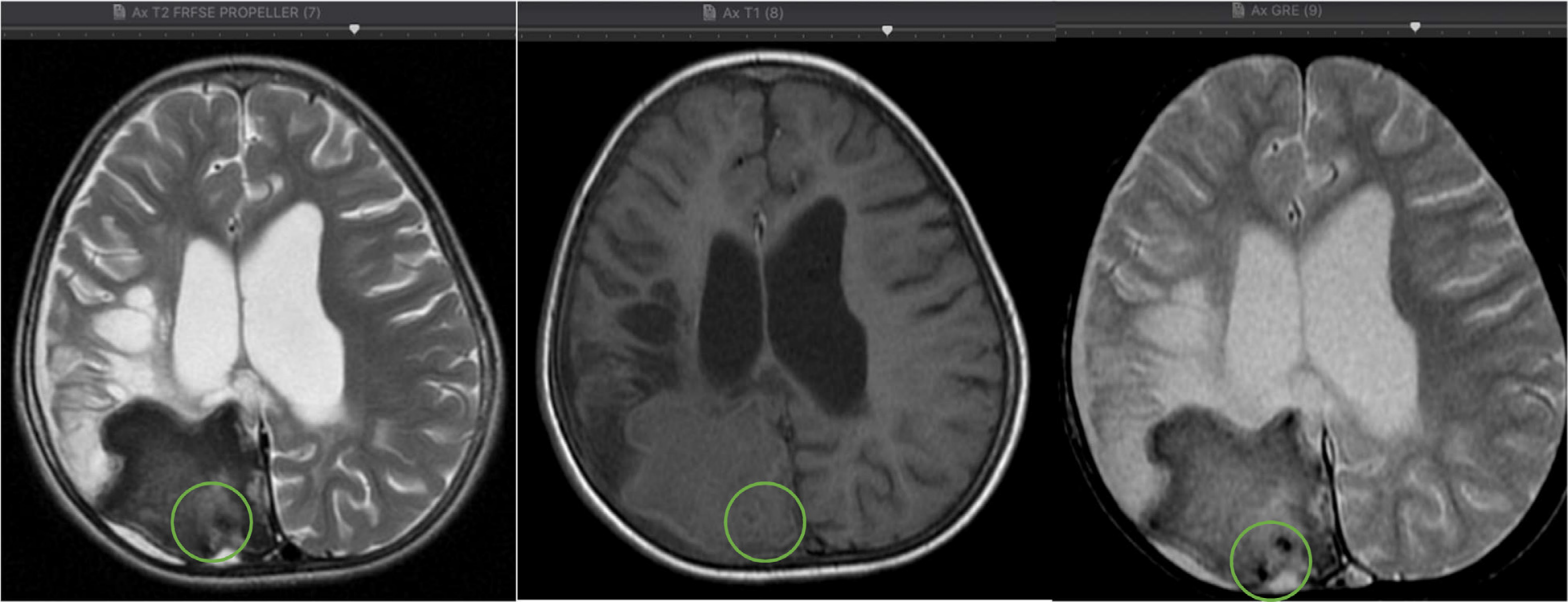
The axial section of the T1, T2 and GRE MRI, Multiple foci calcification in intra- and peri-cystic lesions in the cortex-subcortex of the right occipital lobe (on circle sign).

Another finding from the MRI examination of our case was the presence of tuberculoma in the right cortical-subcortical lobe occipitalis accompanied by a picture of calcification accompanied by a leptomeningeal enhancement in the right temporal-parietal-occipital and the left cisterna basalis as shown in Fig. 4. Tuberculoma is the most common parenchymal lesion in CNS TB that can be solitary or multiple with or without meningitis. Histologically, a mature tuberculoma consists of caseous tissue with central necrosis surrounded by a capsule containing fibroblasts, epithelioid cells, Langerhans giant cells, and lymphocytes. This is also consistent with the findings of the postoperative biopsy results in our case. .

In general, tuberculoma is divided into 2 types, namely caseating and noncaseating granuloma, with MRI examination with contrast playing a vital role in distinguishing between the 2. This difference is produced by the composition of granulation tissue and glial tissue compressed in the central core of the granuloma with MRI examination are described on Table 2 [4,8].

**Table 2:** Differences in granuloma type and tubercular abscess on MRI examination[3].

Sequence	Caseating granuloma (more common)	Noncaseating granuloma	Tubercular Abscess
T1WI	Hypointense	Hypo- to isointense	Hyperintense
T2WI	Hypointense	Hyperintense	Hypointense with hyperintense rim
Edema	+	+	+
Gadolinium (+)	Homogeneous with rim contrast enhancement		

Another important thing to distinguish is whether there is a tubercular abscess because, during contrast administration, both during CT scan and MRI, abscesses produce rim contrast enhancement similar to granulomas. Here, MRI plays an important role: abscesses will present a restricted diffusion area on DWI, while granulomas often show an unrestricted diffusion area. This difference occurs because granulomas contain only a few bacteria, unlike abscesses, whose pus contains numerous cells. Nevertheless, abscesses only appear in 10% of CNS TB cases, especially in immunocompromised and extreme-age patients. Abscesses have a better prognosis and can be treated immediately using surgical drainage [3,9].

Other literature states that tuberculoma is divided into 4 stages, namely noncaseating granuloma, caseating granuloma, caseating granuloma with central liquefaction, and calcified granuloma. Post-treatment, paradoxes in therapeutic response to tuberculoma size may occur. In some cases, it may resolve completely, but most cases often result in calcified granuloma formation. MRI findings may vary but are generally consistent with Table 3 [3].

**Table 3:** MRI findings according to tuberculoma stage [3].

Lesion	T1W	T2W	FLAIR	DWI	T1WCE
Noncaseating granuloma	Iso- to hypointense	Hyperintense	No suppression	No restriction	Homogeneous enhancement
Caseating granuloma	Iso- to hypointense with hyperintense rim	Hypointense	No suppression	No restriction	Homogeneous or ring enhancement
Caseating granuloma with central liquefaction	Isointense to hypointense with hyperintense rim	Hypointense rim with central hyperintensity	Partial suppression	May or may not show restriction	Ring enhancement
Calcified granuloma	Iso- to hypointense	Hypointense	No suppression	No restriction	No enhancement

DWI, diffusion-weighted imaging; FLAIR, Fluid-attenuated inversion recovery; T1WCE: T1W+contrast enhancement.

**Fig.5:**
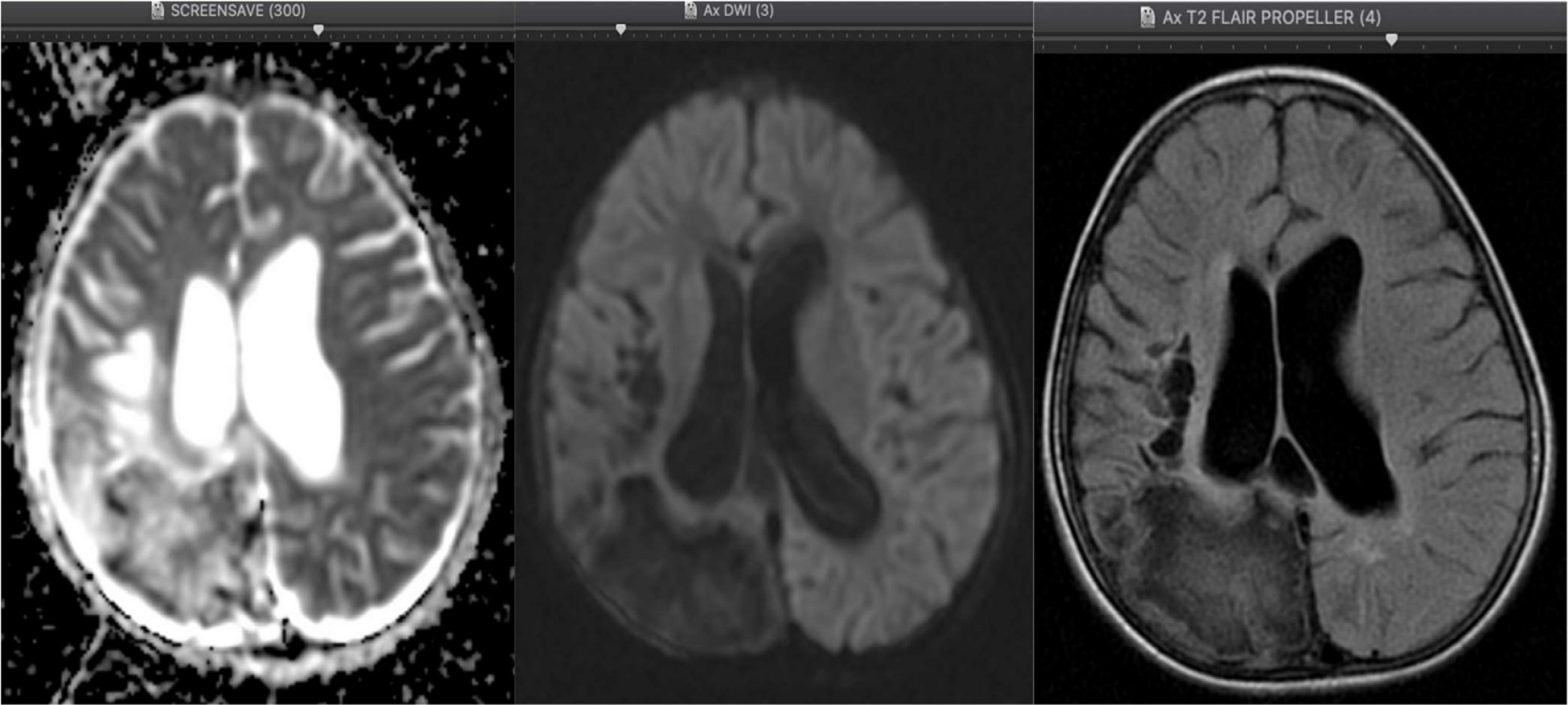
The axial section of the DWI (diffusion-weighted imaging), there is no restricted diffusion area on DWI, no suppression of lesion, and perifocal edema on axial section of the T2 FLAIR (Fluid-attenuated inversion recovery) MRI.

**Fig. 6:**
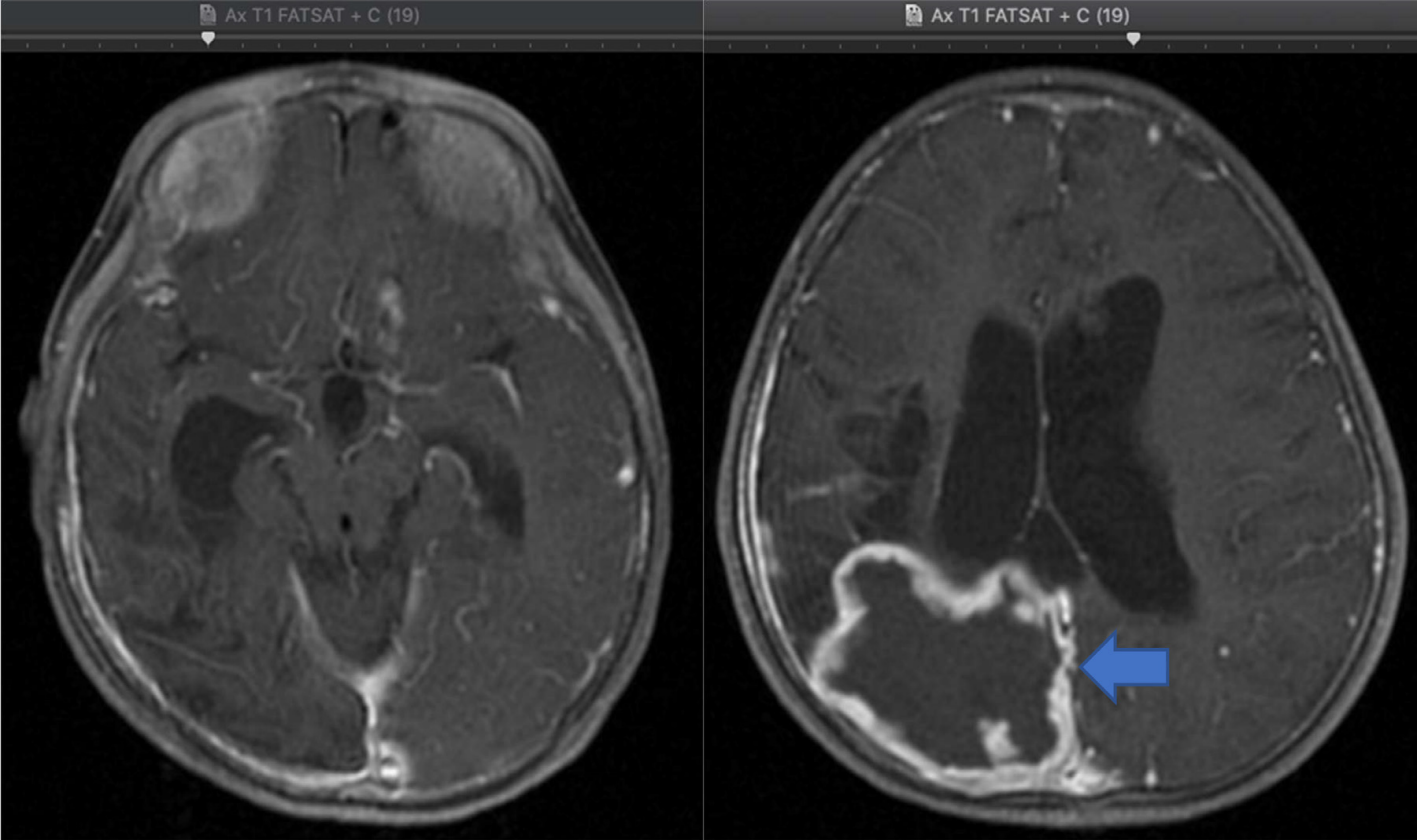
The axial section of the T1 Fatsat with contrast MRI, On intravenous gadolinium contrast administration, pachymeningeal enhancement in the right temporo-parieto-occipital region can be seen, with the enhancement in the left cisterna basalis and rim contrast enhancement in the cortical-subcortical lesion of the right occipital lobe which may represent meningoencephalitis with infected cystic lesion, strongly suggesting tuberculoma (blue arrow).

In this case, MRI and histopathology showed granuloma in the cortical-subcortical right parietal lobe with a suspected caseating phase with *central liquefaction*. Nonetheless, calcified foci were found intra and peri-lesional as seen on Fig. 4, with calcified granuloma excluded due to enhancement during gadolinium contrast administration. The tubercular abscess was the main differential diagnosis of this case; large lesion size (more than 3 cm), firm wall, and localized lesion were other findings that point to an abscess in addition to the presence of rim/ring contrast enhancement. However, the finding of unrestricted diffusion area in Figs. 5 and 6 and the MRI characteristics of the conventional lesions suggested a giant tuberculoma with a caseating granuloma stage accompanied by central liquefaction [3,^9^,10].

**Fig. 7:**
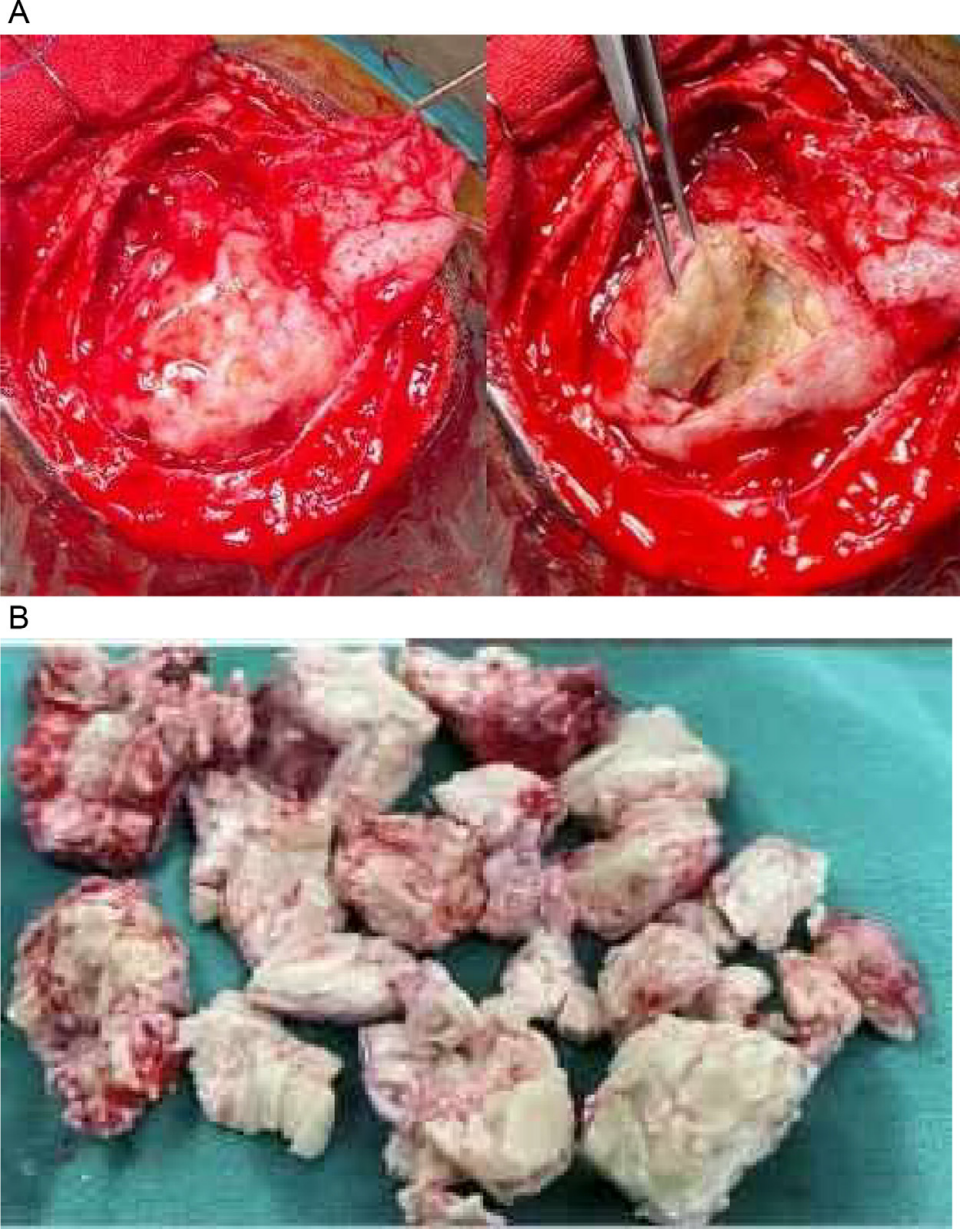
Intraoperative finding of the mass.

**Fig. 8:**
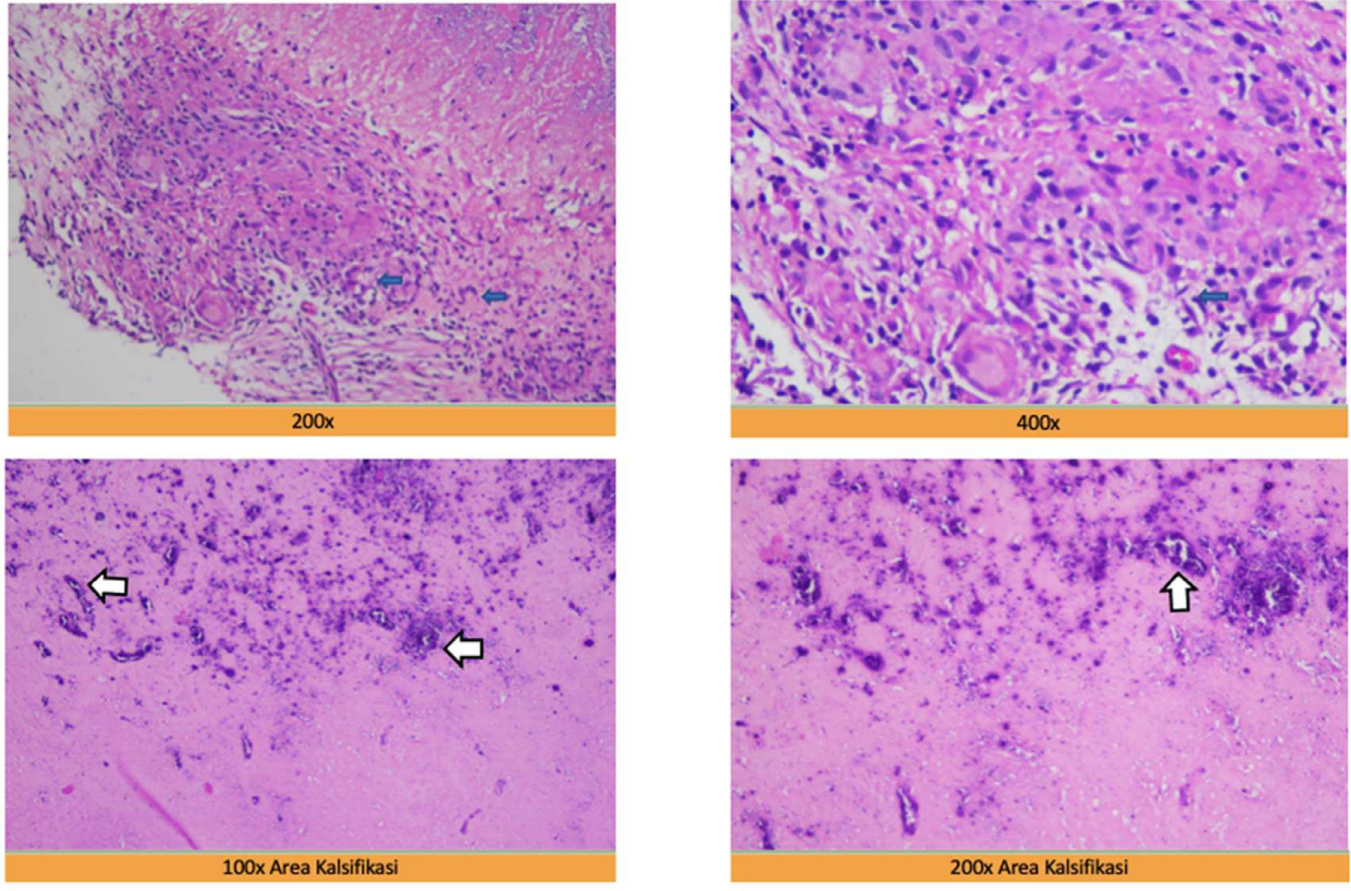
Microscopic examination shows a tissue section which has been stained with hematoxylin and eosin (HE), extensive necrosis in the form of amorphous material with calcification bordered by epithelioid cells arranged in the form of a granuloma (white arrows) and visible Langerhans giant cells (blue arrows), concluded to be granulomatous inflammation suggesting tuberculosis.

The intraoperative clinical findings also agreed that the findings were towards the picture of tuberculoma as shown in Fig. 7, so a wide excision was performed and followed by histopathological examination, as shown in Fig. 8. Common findings in tubercular abscesses are macroscopic abscesses characterized by pus, granulation tissue that forms the abscess wall accompanied by acute and chronic inflammatory cells accompanied by the presence of tubercular bacilli as proven by bacterial culture or acid-resistant bacilli [3].

**Fig. 9:**
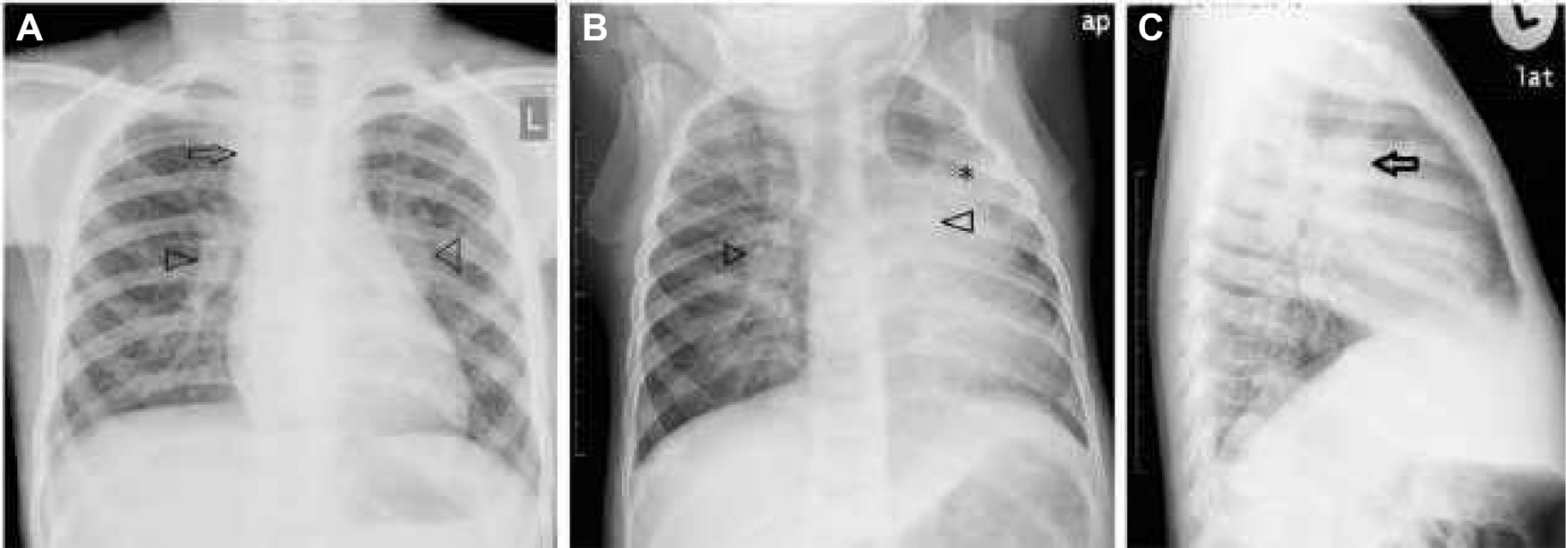
Typical findings in pediatric pulmonary TB, with bilateral hilar lymphadenopathy accompanied by consolidation in the left para-hilar area.

In this case, the histopathology results showed extensive necrotizing tissue pieces in the form of amorphous material with calcification bordered by epithelioid cells arranged in a granuloma pattern, and Langerhans giant cells were seen, consistent with tuberculous granulomatous inflammation. The patient was then treated with antituberculosis and educated about the possibility of sequelae due to CNS TB manifestations by the clinician [1,^11^,12].

## Conclusions

This case report describes the role of MRI in cases of CNS TB, especially with intracranial tuberculoma, with other supporting findings in pediatric cases with TB. MRI can visualize abnormalities with specific characteristics; therefore, clinicians can take appropriate management actions in order to prevent mortality and disability due to sequelae in CNS TB cases. Histopathologic examination is still needed as a means of reconfirmation to differentiate CNS TB cases, especially to rule out the possibility of other infections or neoplasms in the brain parenchyma.

## Patient consent

Written informed consent was obtained from legal guardian of the patient for the publication of this case report.
